# Multi-omics insights into spondyloarthritis and psoriatic arthritis: integrating genomics, transcriptomics, proteomics, and the microbiome for immunological and clinical translation

**DOI:** 10.3389/fimmu.2026.1831823

**Published:** 2026-05-11

**Authors:** Minshun Zhu, Renzhong Li, Sanbing Wu, Jiaping Chen, Kui Sun

**Affiliations:** 1 Department of Rehabilitation, Lu’an Hospital of Traditional Chinese Medicine, Lu’an, China; 2 Department of Orthopedics, Taizhou Hospital of Traditional Chinese Medicine, Taizhou, China; 3 Department of Orthopedics, Second Affiliated Hospital of Anhui University of Traditional Chinese Medicine, Hefei, China

**Keywords:** biomarkers, genomics, microbiome, omics, proteomics, psoriatic arthritis, spondyloarthritis

## Abstract

Spondyloarthritis (SpA) and psoriatic arthritis (PsA) are interrelated, immune-mediated inflammatory diseases characterized by significant clinical heterogeneity. Early differential diagnosis, accurate disease activity assessment, and the development of personalized treatment strategies remain significant clinical challenges. Single-omics approaches have provided only a limited view of this complexity, highlighting the need for integrative strategies. This review systematically synthesizes findings from multi-omics studies—including genomics, transcriptomics, proteomics, and microbiomics—in SpA and PsA. We focus on their application in three key areas: (i) elucidating shared and distinct immunopathological mechanisms, (ii) facilitating differential diagnosis, and (iii) discovering novel biomarkers. By comparing their molecular landscapes, we explore the shared and distinct immunological foundations of SpA and PsA. Furthermore, we critically evaluate the translational potential of integrated multi-omics strategies for advancing early diagnosis, precision monitoring, predicting treatment responses, and identifying novel therapeutic targets. This integrated, multi-omics framework promises to refine disease taxonomy and guide personalized therapeutic decisions, paving the way for precision medicine in SpA and PsA.

## 1 Introduction

Spondyloarthritis (SpA) represents a family of interrelated, immune-mediated inflammatory diseases unified by shared clinical and genetic features. Psoriatic arthritis (PsA) is a distinct and major subtype within the SpA spectrum, alongside ankylosing spondylitis (AS), non-radiographic axial SpA (nr-axSpA), reactive arthritis, and enteropathic arthritis. These conditions share common immunopathological pathways, most notably the IL-23/IL-17 axis, and can present with overlapping musculoskeletal manifestations, including axial involvement (sacroiliitis and spondylitis), peripheral arthritis, enthesitis, and dactylitis. However, the relative frequency and prominence of these features vary substantially across subtypes. They exhibit significant differences in genetic susceptibility, skin manifestations, joint involvement patterns, and disease progression (1). Global epidemiological data reveal substantial variation in SpA and PsA prevalence across populations. For instance, axial spondyloarthritis (axSpA) is most prevalent in circumpolar Arctic populations and lowest in Japanese and African American populations (2). These epidemiological differences, alongside the incomplete penetrance of even the strongest risk factor, *HLA-B*27* (2, 3), underscore the complex interplay between genetic background and environmental factors, providing a strong rationale for multi-omic investigation.

Traditional diagnosis relies on clinical and imaging criteria, often resulting in delayed diagnosis and a lack of precise biomarkers to predict treatment response. For example, among patients presenting to chiropractic clinics for chronic back pain, the ASAS referral strategy identified undiagnosed SpA in over 12% of cases, with non-radiographic axSpA being the most common (4). This underscores the importance of early identification and referral in primary care and specialty clinics. Furthermore, diagnosing PsA is particularly challenging, especially in cases without cutaneous psoriasis (PsA sine psoriasis), where differential diagnosis overlaps with peripheral SpA (pSpA). Although both share similarities in age of onset, number of affected joints, and prevalence of axial involvement, pSpA predominantly affects males and exhibits a higher prevalence of *HLA-B*27*, enthesitis, and large lower limb joint involvement compared to PsA sine psoriasis. Notably, the prevalence of *HLA-B*27* varies markedly across the SpA spectrum, being most strongly associated with axial disease (present in 80–95% of patients with AS) and less frequent in predominantly peripheral forms. Conversely, PsA sine psoriasis shows higher prevalence of *HLA-C*06:02*, dactylitis, and distal interphalangeal joint involvement in the hands. These differences indicate that the distinction between pSpA and PsA sine psoriasis extends beyond semantics, necessitating improved disease characterization (5).

In recent years, the emergence of multi-omics technologies has provided powerful tools for analyzing complex diseases at the systems level. Integrating genomics, proteomics, and microbiomics data enables comprehensive mapping of disease-associated molecular networks, revealing core molecular differences and commonalities between SpA and PsA. This approach facilitates the discovery of highly specific and sensitive biomarker combinations (6). For instance, emerging omics layers such as metabolomics promise to provide even deeper insights into the functional consequences of host–microbe interactions in SpA pathogenesis, representing a critical area for future multi-omic integration (7). In multi-omics studies of PsA and psoriasis (PsO), integrating genomic, transcriptomic, epigenetic, proteomic, metabolomic, and microbiome data helps elucidate disease distinctions, broadens our understanding of pathogenesis, and provides valuable biomarker insights for early diagnosis and treatment (6). Furthermore, single-cell RNA sequencing (scRNA-seq) has transformed our understanding of immune-mediated arthritis, including rheumatoid arthritis and SpA. It resolves the heterogeneity of synovial fibroblast and immune cell subpopulations, offering insights into disease mechanisms and therapeutic response variations. In SpA, particularly PsA and ankylosing spondylitis (AS), scRNA-seq studies have identified distinct cellular landscapes associated with disease pathology (8).

**Figure 1:**
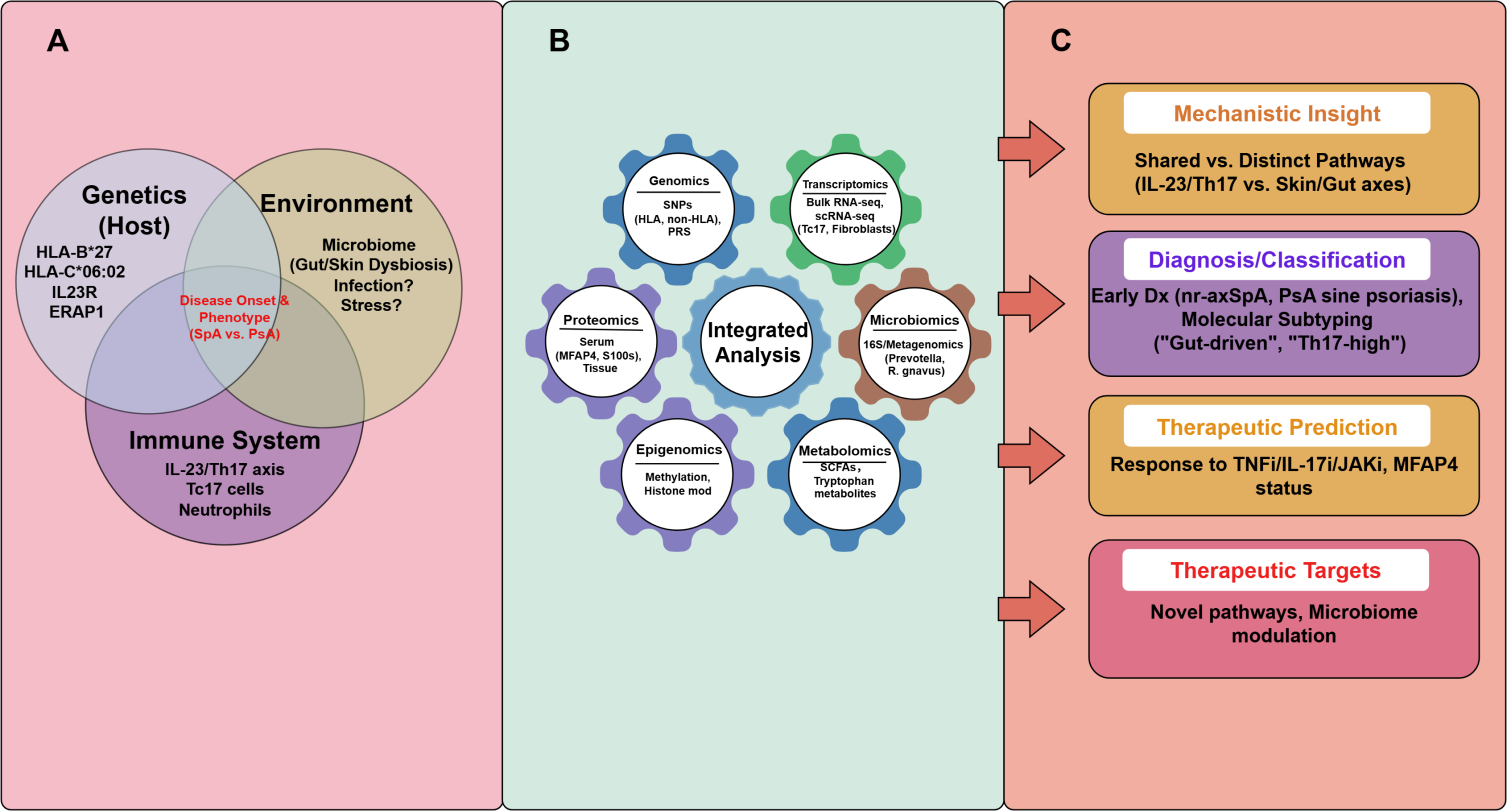
The multi-omics landscape of SpA and PsA: from risk to translation. This schematic provides a conceptual framework for integrating multi-omics data in spondyloarthritis (SpA) and psoriatic arthritis (PsA). **(A)** Illustrates the interplay of genetic susceptibility (e.g., *HLA-B*27*, *HLA-C*06:02*), environmental factors (e.g., microbiome dysbiosis), and immune dysregulation (e.g., IL-23/Th17 axis) converging to drive disease onset and phenotype. **(B)** Depicts the diverse omics layers—genomics, transcriptomics, proteomics, epigenomics, metabolomics, and microbiomics—that are integrated through computational approaches to construct a systems-level understanding. **(C)** Summarizes the translational outputs of this integration: mechanistic insights into shared and distinct pathways, improved diagnostic classification, prediction of therapeutic responses, and identification of novel treatment targets. This framework underscores the paradigm shift from single-omics approaches toward integrated precision medicine in SpA and PsA. SpA, spondyloarthritis; PsA, psoriatic arthritis; PRS, polygenic risk score; scRNA-seq, single-cell RNA sequencing; MFAP4, microfibrillar-associated protein 4; SCFAs, short-chain fatty acids; nr-axSpA, non-radiographic axial spondyloarthritis; TNFi, tumor necrosis factor inhibitors; IL-17i, interleukin-17 inhibitors; JAKi, Janus kinase inhibitors. Figure drawn by www.figdraw.com.

As summarized in **Figure 1**, the integration of genomics, transcriptomics, proteomics, and microbiomics offers a systems-level perspective that can refine disease taxonomy and guide personalized therapeutic decisions—a central theme of this review. This not only deepens our understanding of the immunopathogenesis of diseases but also drives the transformation of clinical practice toward early intervention and personalized treatment. Precision medicine, propelled by advances in multi-omics approaches and analytics, aims to revolutionize patient care by using clinically actionable molecular biomarkers to guide diagnostic and therapeutic decisions (9). In PsA, stratified therapy targeting specific biologics demonstrates potential for high treatment response, signaling the promise of precision medicine (10, 11). However, reliable biomarkers to predict treatment response in chronic inflammatory diseases (CIDs) remain elusive. Studies indicate that elevated serum microfibrillar-associated protein 4 (MFAP4) status prior to initiating biologic therapy correlates with favorable clinical outcomes (12). Furthermore, in-depth characterization of synovial tissue has provided new insights into the diverse cellular and molecular features of these diseases and their potential links to different clinical and therapeutic response phenotypes. This advancement raises the prospect of improving response rates by matching specific drug use with homologous targeted pathways that may drive specific disease subtypes in particular patient populations. Achieving this goal requires innovative, patient-centered, molecular pathology-driven clinical trial approaches (13).

### 1.1 Review scope and search strategy

This is a narrative review aimed at synthesizing and critically evaluating key findings from multi-omics studies in SpA and PsA. We performed a comprehensive literature search of the PubMed and Web of Science databases for articles published in English up to March 2026. Search terms included combinations of ‘spondyloarthritis’, ‘ankylosing spondylitis’, ‘psoriatic arthritis’, with ‘genomics’, ‘GWAS’, ‘transcriptomics’, ‘single-cell RNA sequencing’, ‘proteomics’, ‘microbiome’, ‘multi-omics’, and ‘biomarker’. This review focuses on the integration of genomics, transcriptomics, proteomics, and microbiomics, as these layers currently offer the most robust and clinically relevant data for SpA and PsA. While epigenomics and metabolomics represent promising frontiers, their systematic multi-omic integration in these specific diseases remains nascent and is thus discussed primarily as key future directions (Section 8). Given the narrative nature of this review, the selection of studies was based on their relevance to the overarching themes, and as such, it may be subject to selection bias. A formal systematic review with pre-defined inclusion/exclusion criteria was not conducted.

## 2 The genomic landscape of SpA and PsA: from genetic susceptibility to functional interpretation

### 2.1 Shared and specific genetic risk loci

The genetic architecture of spondyloarthritis (SpA) and psoriatic arthritis (PsA) exhibits both significant overlap and key differences, providing crucial clues for understanding their shared immunopathological mechanisms and distinct clinical presentations. Differences in association with major histocompatibility complex (MHC) region alleles, particularly human leukocyte antigen (HLA) variants, are especially pronounced. *HLA-B*27* represents the strongest genetic risk factor for ankylosing spondylitis (AS, the predominant SpA subtype), with its association confirmed across multiple populations including Chinese cohorts (14). However, the association of *HLA-B*27* with PsA is relatively weak, whereas polymorphisms at the *HLA-C* locus show significant correlation with PsA risk. A meta-analysis of populations of European and Middle Eastern descent revealed that the *HLA-C*06:02* allele is strongly associated with increased PsA risk, while *HLA-C*04* may confer protective effects (15). *HLA-C*06:02* is also a major genetic determinant of psoriatic skin manifestations, and its association with PsA arthritis subtypes suggests a potential shared immunogenetic basis between skin and joint lesions. Additionally, *HLA-B*27* and *HLA-B*38* alleles are considered potential biomarkers for PsA (16).

In non-MHC regions, SpA and PsA share multiple susceptibility genes, collectively pointing to the central role of the IL-23/Th17 inflammatory axis. For instance, the IL23R and IL12B loci represent key shared risk sites for both diseases (17). Variants in these genes may disrupt IL-23 signaling pathways, thereby driving abnormal Th17 cell activation and proinflammatory cytokine production (e.g., IL-17), forming the shared immunogenetic basis for both diseases (18). Furthermore, variations in genes such as TNFAIP3 (encoding the TNIP1 protein) are also associated with PsA susceptibility, further underscoring the importance of immunoregulatory pathways in disease pathogenesis (19). Beyond adaptive immune genes, those involved in innate immunity are also linked to PsA risk, suggesting an autoinflammatory disease mechanism in at least a subset of patients (20).

**Figure 2:**
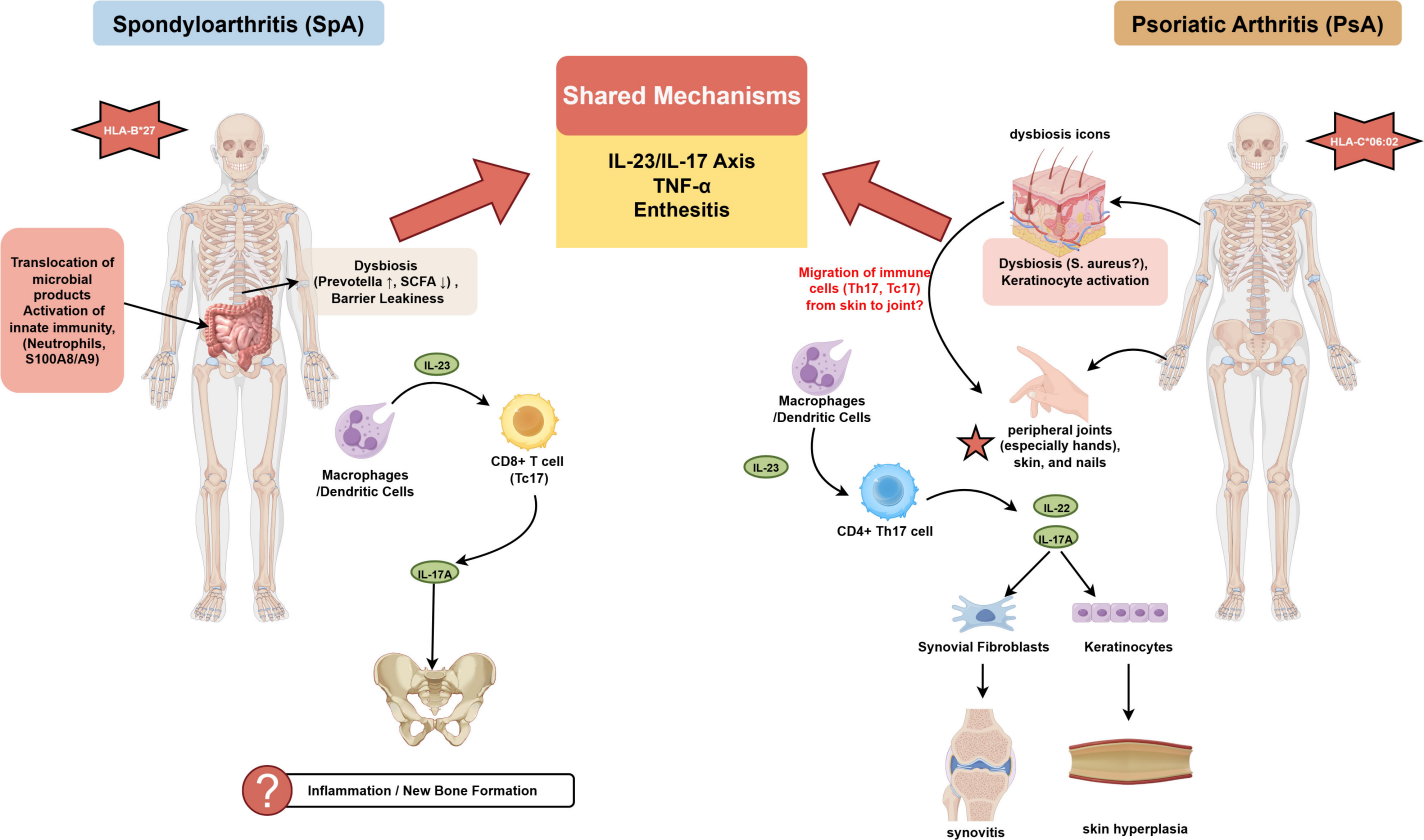
Shared and distinct immunopathological mechanisms in SpA and PsA. This schematic compares the key pathogenic pathways driving spondyloarthritis (SpA, left) and psoriatic arthritis (PsA, right). In SpA, the strong genetic association with *HLA-B*27* and prominent gut dysbiosis (Prevotella enrichment, reduced SCFA-producing bacteria) support the “mucosal origin” hypothesis. Gut barrier disruption enables translocation of microbial products, activating innate immune cells (neutrophils, S100 alarmins) and driving IL-23/IL-17 axis activation, particularly via IL-17A-producing CD8^+^ T cells (Tc17), culminating in axial and entheseal inflammation. In PsA, the genetic architecture is defined by *HLA-C*06:02*, with a more prominent role for skin dysbiosis and keratinocyte activation, suggesting a “skin-joint axis.” Immune activation involves both CD4^+^ Th17 and CD8^+^ Tc17 cells, with IL-17 driving synovial fibroblast activation and keratinocyte hyperplasia, manifesting as peripheral synovitis, dactylitis, and psoriatic skin lesions. The central overlapping region highlights shared mechanisms—the IL-23/IL-17 axis, TNF-α signaling, and enthesitis—that unite these diseases while their distinct genetic and tissue-specific immune dysregulations underlie divergent clinical phenotypes. SCFAs, short-chain fatty acids; IL, interleukin; Th17, T helper 17; Tc17, IL-17-producing CD8^+^ T cell. Figure drawn by www.figdraw.com.

Despite numerous shared loci, studies have also identified relatively specific genetic signals for PsA, which help explain its unique clinical heterogeneity. For example, genes such as RUNX3 and PTPN22 are considered relatively specific genetic signals for PsA (17). These genes may drive the unique joint and skin manifestations of PsA through pathways such as influencing T cell function and regulating T cell (Treg) activity. A study of Italian PsA patients found that polymorphisms in genes such as TRAF3IP2, ERAP1, and STAT4 were associated with disease susceptibility. Variants in TRAF3IP2 were additionally linked to higher joint tenderness/swelling counts and disease activity. Multigene risk analysis further indicated that individuals carrying at least four risk alleles had a higher probability of developing PsA (19, 20). These findings underscore the significant role of non-HLA genes in PsA pathogenesis and suggest that the genetic background of PsA may involve subtle imbalances in T cell activation and immune regulation. The key shared and distinct genetic risk loci discussed above are summarized in **Table 1**. Building on this genetic landscape, **Figure 2** provides a visual synthesis of the immune mechanisms differentiating SpA from PsA, serving as a foundation for the subsequent discussion on functional genomics and biomarker discovery.

**Table 1:** Key shared and distinct genetic risk loci in spondyloarthritis and psoriatic arthritis.

Genetic locus	Association in SpA	Association in PsA	Functional implications	References
*HLA-B*27*	Strongest risk factor for AS; confirmed across multiple populations including Chinese cohorts	Relatively weak association	Antigen presentation; may predispose to gut dysbiosis and altered immune responses	(14–16)
*HLA-C*06:02*	Weak or no association	Strong risk factor for PsA; also major determinant of psoriatic skin manifestations	Antigen presentation; potential shared immunogenetic basis between skin and joint lesions	(15, 16)
*HLA-C*04*	Not reported	Protective effect against PsA	Unknown	(15)
*HLA-B*38*	Not reported	Potential biomarker for PsA	Unknown	(16)
*IL23R*	Shared risk locus	Shared risk locus	IL-23 signaling; Th17 cell differentiation and stabilization	(17, 18)
*IL12B*	Shared risk locus	Shared risk locus	IL-12/IL-23 p40 subunit; Th1/Th17 polarization	(17)
*TNFAIP3 (TNIP1)*	Associated with AS	Associated with PsA	Negative regulator of NF-κB signaling; immunoregulation	(19)
*RUNX3*	Not specifically reported	Relatively specific genetic signal for PsA	T cell development and function; CD8^+^ T cell differentiation	(17)
*PTPN22*	Not specifically reported	Relatively specific genetic signal for PsA	T cell receptor signaling; regulatory T cell function	(17)
*TRAF3IP2*	Associated with AS	Associated with PsA; linked to higher joint counts and disease activity	IL-17 signaling adaptor; NF-κB activation	(19, 20)
*ERAP1*	Associated with AS	Associated with PsA	MHC class I peptide processing; *HLA-B*27* interaction	(19, 20)
*STAT4*	Associated with AS	Associated with PsA	Th1/Th17 differentiation; IL-12/IL-23 signaling	(19, 20)

This table consolidates evidence from genome-wide association studies and meta-analyses, demonstrating the shared and distinct genetic architectures of SpA and PsA, with emphasis on HLA and non-HLA loci that inform immunological pathways and disease heterogeneity.

AS, ankylosing spondylitis; HLA, human leukocyte antigen; IL, interleukin; MHC, major histocompatibility complex; NF-κB, nuclear factor kappa-B; PsA, psoriatic arthritis; SpA, spondyloarthritis; Th, T helper.

### 2.2 Functional translation of genomic data and mechanistic exploration

Genome-wide association studies (GWAS) have successfully identified numerous genetic risk loci associated with SpA and PsA. However, translating these statistical associations into biological understanding of disease mechanisms remains a central challenge. Functional genomics approaches offer powerful tools for this purpose. By integrating expression quantitative trait locus (eQTL) analysis—which links genetic variants to gene expression levels—with chromatin conformation capture techniques, researchers can map non-coding risk variants identified in GWAS to specific target genes and cell types they regulate (21). For instance, these methods help reveal how risk variants influence gene expression in specific cell types—such as CD8+ T cells and intestinal epithelial cells—through distant enhancer-promoter interactions, thereby elucidating their role in disease pathogenesis. This multi-layered analytical strategy—encompassing eQTL, methylation, and chromatin conformation studies—is crucial for identifying novel genes affected by disease-associated variants (22).

While epigenetic modifications—including DNA methylation, histone modifications, and non-coding RNA regulation—serve as critical bridges between genetic susceptibility and environmental triggers in SpA and PsA (23), a comprehensive multi-omics review of this layer is beyond the scope of the current work. Large-scale, integrated epigenomic studies specifically in SpA and PsA are still in their infancy, and robust comparative data across disease subtypes remain limited. Nevertheless, the potential of epigenetic markers as biomarkers and therapeutic targets represents a crucial avenue for future investigation, as discussed in Section 8.2.

Integrating multiple genetic risk loci into polygenic risk scores (PRS) represents a key direction for translating genomic data into clinical practice. PRS quantify an individual’s genetic susceptibility burden and show promise in assessing disease risk and predicting severity (24). For instance, studies have attempted to use PRS incorporating both HLA and non-HLA loci to distinguish patients with psoriasis alone from those with concomitant PsA (25). However, current PRS models exhibit limited discriminatory power, posing challenges to their clinical utility (26). These challenges stem from several sources: First, the number of identified PsA-specific loci remains limited, necessitating larger-scale studies and international collaboration to uncover additional genetic factors (17). Second, PRS performance may vary across different races and ethnic groups, requiring consideration of genetic background diversity (27). Finally, integrating PRS with clinical risk factors to construct more robust risk prediction models requires further exploration and validation (17). Nevertheless, utilizing PRS for disease risk stratification to enable early intervention and precision medicine remains a promising direction in this field (28).

## 3 Transcriptomics and proteomics: unveiling dynamic immune and inflammatory pathways

### 3.1 Transcriptomic profiling in peripheral blood and lesion tissue

Transcriptomic profiling of peripheral blood and affected tissues has revealed distinct immune landscapes in SpA and PsA. For example, synovial fluid from early SpA patients is characterized by an expansion of IL-17A^+^ CD8^+^ T (Tc17) cells, a finding that distinguishes them from patients with rheumatoid arthritis (29). Although these diseases, including ankylosing spondylitis (AS) and PsA, share strong genetic backgrounds such as *HLA-B*27*, they exhibit distinct clinical phenotypes. This heterogeneity stems partly from differences at the level of gene regulation (30). By comparing the transcriptomes of peripheral blood mononuclear cells (PBMCs) from patients, eQTL studies help uncover disease-specific gene expression regulatory networks. For instance, in PsA, specific genetic variants may influence the expression activity of the type I interferon signaling pathway or cytotoxic-related gene modules via eQTL mechanisms, thereby driving immune responses associated with psoriatic skin lesions. This contrasts with the transcriptional characteristics of AS, which primarily involves axial skeletal involvement (31). This analysis of transcriptome differences based on genetic background provides a molecular foundation for understanding divergent immune pathway activation between the two diseases.

Transcriptomic studies of affected tissues—such as joint synovium, psoriatic skin lesions, and intestinal mucosa—reveal the specificity of local inflammatory microenvironments. eQTL analysis can identify genetic regulatory elements acting in these specific tissues, thereby elucidating the genetic basis for tissue-specific inflammatory features (31, 32). For instance, in synovial tissue from SpA patients, eQTLs may regulate gene expression associated with myeloid cell infiltration and stromal cell activation, shaping an inflammatory environment rich in fibroblasts and macrophages (33). In contrast, within PsA skin lesions, eQTLs may preferentially influence the regulation of genes associated with keratinocyte proliferation and differentiation, leading to their highly activated state (31, 34). These tissue-specific eQTL effects translate shared genetic risk factors into distinct pathological manifestations across target organs, elucidating potential mechanisms underlying the clinical diversity of SpA and PsA.

Single-cell RNA sequencing (scRNA-seq) has further resolved this heterogeneity by identifying disease-relevant cell subpopulations, such as pathogenic Th17 cells, cytotoxic CD8^+^ T cells, and activated fibroblasts, at single-cell resolution (8). Integration of scRNA-seq with genetic data can now map how disease-associated variants influence gene expression within these specific cell types (31). By resolving the heterogeneity of immune cells and stromal cells in tissues such as synovium and skin, scRNA-seq identifies active eQTLs within specific cell subpopulations (e.g., Th17 cells, cytotoxic CD8+ T cells, innate lymphoid cells type 3). This cell-type-specific eQTL information is crucial for constructing precise intercellular communication networks. For example, an eQTL discovered in CD8+ T cells may regulate the expression of their cytotoxic-related genes, thereby influencing their ability to attack synovial or skin tissues (31, 35). Integrating scRNA-seq data with genetic information enables the mapping of disease-associated genetic variants regulating gene expression within specific cell types. This not only deepens our understanding of the immunopathological mechanisms underlying SpA and PsA but also provides new leads for discovering cell-targeted therapeutic strategies.

### 3.2 Proteomic signatures in serum and synovial fluid: from candidate biomarkers to clinical utility

Proteomic technologies, including mass spectrometry and multiplex immunoassays, have enabled the identification and quantification of a range of candidate protein biomarkers in the serum and synovial fluid of patients with SpA and PsA. These efforts aim to capture the dynamic state of inflammation and tissue remodeling in a minimally invasive manner.

Among the most extensively studied candidates is Matrix Metalloproteinase-3 (MMP-3), an enzyme involved in extracellular matrix degradation. Elevated serum MMP-3 levels have been consistently associated with radiographic progression in ankylosing spondylitis (AS) and with active synovitis in PsA, suggesting its utility as a marker of structural damage and disease activity (36). Similarly, the alarmins S100A8 and S100A9, which form the calprotectin heterodimer, are released by activated neutrophils and monocytes. Their concentrations in serum and synovial fluid correlate strongly with clinical disease activity indices such as the Ankylosing Spondylitis Disease Activity Score (ASDAS) and the Disease Activity in Psoriatic Arthritis (DAPSA) score, reflecting the intensity of innate immune cell activation in both SpA and PsA (37). More recently, Microfibrillar-Associated Protein 4 (MFAP4) has emerged as a promising predictive biomarker; elevated baseline serum MFAP4 levels prior to biologic therapy initiation have been associated with a favorable clinical response in chronic inflammatory diseases, including SpA and PsA (12).

Beyond the serum compartment, proteomic analysis of synovial fluid (SF) offers a direct window into the local joint microenvironment. Comparative SF proteomics has revealed distinct molecular signatures that differentiate SpA from other inflammatory arthritides such as rheumatoid arthritis (RA). For instance, SF from SpA patients may exhibit a unique profile enriched in proteins related to neutrophil degranulation and the complement cascade, whereas RA SF shows a signature more closely linked to adaptive immune responses and B cell activation (38).

The clinical utility of these proteomic findings is significantly enhanced when interpreted through a genetic lens. Integrating proteomics with expression quantitative trait locus (eQTL) analysis provides a framework for distinguishing correlative inflammatory markers from proteins with a potentially causal role in pathogenesis (31). For example, if elevated levels of a candidate protein such as S100A9 are associated with a specific eQTL known to regulate its gene expression, this strengthens the evidence for a genetically driven, pathogenic role rather than a mere secondary inflammatory phenomenon. This multi-omics integration strategy improves biomarker discovery accuracy and clinical translational value by prioritizing candidates with robust genetic support.

In the context of differential diagnosis, identifying protein signatures that distinguish PsA from cutaneous psoriasis alone (PsO) or from other forms of SpA remains a key challenge. eQTL-guided proteomics can aid this effort by pinpointing protein expression patterns that are specifically activated in the PsA disease context (5, 31, 39). Systematic screening of these genetically regulated, differentially expressed proteins holds promise for constructing biomarker panels with high discriminatory power.

For treatment prediction, baseline proteomic profiles are being actively investigated for their ability to forecast responses to biologics such as TNF inhibitors and IL-17 inhibitors (31, 40). The predictive power of these protein markers can be further refined by considering the underlying eQTL genotypes that regulate their expression (41). For example, an eQTL associated with the expression of a key component in the IL-17 signaling pathway could help identify patients more likely to respond to IL-17 blockade (31). Such approaches not only facilitate personalized therapeutic decisions but also provide mechanistic insights into the biological determinants of drug response, advancing the application of precision medicine in SpA and PsA. **Table 2** consolidates the key transcriptomic and proteomic findings from peripheral blood, synovial fluid, and affected tissues, demonstrating the distinct immune cell landscapes and protein biomarkers that characterize SpA and PsA and their potential in diagnosis and treatment prediction.

**Table 2:** Transcriptomic and proteomic findings in SpA and PsA.

Biomarker/cell type	Sample type	Finding in SpA	Finding in PsA	Clinical relevance	References
Tc17 cells (IL-17A^+^ CD8^+^ T cells)	Synovial fluid	Significantly expanded in early SpA; distinguishes from RA	Also expanded; implicated in pathogenesis	Diagnostic marker for early inflammatory arthritis; therapeutic target	(8, 29)
CD8^+^ IFNγ^+^ T cells	Synovial fluid	Not dominant	Not dominant	Dominant in RA, aiding differential diagnosis	(29)
Type I interferon signaling	PBMCs	Not prominently featured	eQTL-driven activation associated with skin lesions	Distinct immune pathway activation between diseases	(31)
Cytotoxic gene modules	PBMCs	Not prominently featured	eQTL-driven activation	May drive psoriatic skin and joint manifestations	(31)
Myeloid cell/stromal genes	Synovial tissue	eQTLs regulate genes associated with myeloid infiltration and stromal activation	May also be involved	Shapes inflammatory microenvironment rich in fibroblasts and macrophages	(33)
Keratinocyte proliferation genes	Skin lesions	Not applicable	eQTLs regulate genes associated with keratinocyte proliferation and differentiation	Drives psoriatic skin phenotype	(31, 34)
Pathogenic Th17 cells	Synovium/skin	Identified by scRNA-seq	Identified by scRNA-seq	Disease-relevant cell subpopulation; therapeutic target	(8)
Activated fibroblasts	Synovium	Identified by scRNA-seq	Identified by scRNA-seq	Contribute to synovial inflammation and joint damage	(8)
MMP-3	Serum/synovial fluid	Elevated levels associated with radiographic progression in AS and active synovitis in PsA	Elevated levels associated with active synovitis	Marker of structural damage and disease activity	(36)
S100A8/A9 (calprotectin)	Serum/synovial fluid	Concentrations correlate strongly with ASDAS	Concentrations correlate strongly with DAPSA	Reflects innate immune cell activation; correlates with disease activity	(37)
MFAP4	Serum	Elevated baseline levels correlate with favorable response to biologics	Elevated baseline levels correlate with favorable response to biologics	Predictive biomarker for treatment response	(12)
Synovial fluid proteome	Synovial fluid	Enriched in neutrophil degranulation and complement cascade proteins	Not specifically detailed	Differentiates SpA from RA (which shows adaptive/B cell signature)	(38)

This table consolidates evidence from bulk and single-cell transcriptomic studies alongside proteomic analyses, demonstrating the distinct immune cell landscapes and protein biomarkers that characterize SpA and PsA and their potential in diagnosis and treatment prediction.

eQTL, expression quantitative trait locus; IFNγ, interferon-gamma; IL, interleukin; MFAP4, microfibrillar-associated protein 4; MMP-3, matrix metalloproteinase-3; PBMCs, peripheral blood mononuclear cells; PsA, psoriatic arthritis; RA, rheumatoid arthritis; scRNA-seq, single-cell RNA sequencing; SpA, spondyloarthritis; Tc17, IL-17-producing CD8+ T cell; Th, T helper.

## 4 Gut and skin microbiomes: key interfaces for environmental triggering and immune regulation

### 4.1 Gut dysbiosis and spondyloarthritis

The gut microbiome of patients with spondyloarthritis (SpA) exhibits characteristic dysbiosis. Multiple metagenomic studies confirm that compared to healthy controls, SpA patients show significantly reduced gut microbial diversity, a decrease in biodiversity similar to findings in inflammatory bowel disease (IBD) (42). Regarding specific microbial composition, patients with ankylosing spondylitis (AS) exhibit enrichment of *Prevotella* spp. and reduced *Bacteroides* (43). A study of Han Chinese AS patients further revealed increased levels of potentially harmful bacteria such as Proteobacteria and Enterobacteriaceae in the gut, alongside reduced levels of beneficial bacteria like Firmicutes and Actinobacteria (44). This dysbiosis correlates closely with disease activity. Studies indicate that gut dysbiosis is more prevalent in AS patients than in healthy controls and is significantly associated with poorer disease activity (e.g., higher ASDAS-CRP and BASDAI scores) and worse physical function (BASFI), an association that appears independent of gut inflammation and treatment factors (45). Furthermore, the abundance of specific bacteria correlates with disease activity. For instance, *Ruminococcus gnavus* abundance increases in SpA patients and ranks among the top species distinguishing patients from healthy controls (46). *Coprobacter* spp. abundance also positively correlates with BASDAI and ASDAS-CRP, suggesting its potential as a disease activity biomarker (47). Genetic background, particularly *HLA-B*27*, plays a pivotal role in shaping the gut microbiota. Studies reveal that even among healthy siblings of SpA patients, *HLA-B*27*-positive individuals exhibit significantly different gut microbiota composition compared to *HLA-B*27*-negative individuals (46). *HLA-B*27* expression alone can significantly alter gut microbiota composition in mice, driving the microbial ecosystem toward a pro-inflammatory state (48). Importantly, gut dysbiosis is not restricted to axial SpA. Studies in psoriatic arthritis (PsA) have also demonstrated characteristic alterations in the gut microbiome, including reduced diversity and shifts in specific bacterial taxa, which may contribute to the systemic inflammatory burden in these patients (49, 50).

Gut dysbiosis profoundly impacts host immune homeostasis through altered metabolic products, thereby contributing to SpA pathogenesis. Short-chain fatty acids (SCFAs) are important anti-inflammatory metabolites produced by beneficial gut bacteria fermenting dietary fiber. Studies reveal that SCFA-producing bacteria (e.g., Megamonas and Lachnoclostridium) are significantly reduced in the intestines of AS patients, with their abundance negatively correlated to disease severity (51). SCFA depletion weakens their role in maintaining intestinal barrier integrity and their ability to induce regulatory T cells (Tregs). Concurrently, the tryptophan metabolic pathway undergoes significant alterations in SpA patients. Metabolomic analysis of colon biopsy specimens from axial SpA (axSpA) patients revealed substantial amplification of tryptophan metabolites such as indole-3-acetic acid (IAA) and indole-3-acetaldehyde (I3Ald). while corresponding metagenomic analysis showed increased abundance of microbial genes producing these metabolites (e.g., indolepyruvate decarboxylase genes) in axSpA patients (52). These metabolic alterations may influence both local and systemic immune responses. For instance, reduced levels of butyrate (an SCFA) and butyrate-producing bacteria like *Faecalibacterium prausnitzii* disrupt the Th17/Treg cell balance. Experiments demonstrate that introducing F. prausnitzii or its metabolite butyrate into CD4+ T cells from axSpA patients reduces IL-17A production and increases IL-10 levels, while butyrate also diminishes osteoclastogenesis (53). Furthermore, altered gut metabolic profiles in *HLA-B*27* transgenic mice manifest as elevated levels of multiple prostaglandins and reduced levels of anti-inflammatory metabolites (48). These metabolic dysregulations collectively compromise intestinal barrier function, increasing intestinal permeability. This facilitates translocation of microbial products (e.g., lipopolysaccharides) into the systemic circulation, where they activate innate immunity via pattern recognition receptors and drive adaptive immune responses such as the IL-23/IL-17 axis, ultimately leading to joint inflammation (54, 55).

Given the critical role of the gut microbiome in SpA pathogenesis, microbiome-targeting interventions demonstrate clinical potential. Antibiotic interventions can influence disease progression. In *HLA-B*27* transgenic rats, broad-spectrum antibiotic treatment restored intestinal sclerotin expression and normalized serotonin synthesis, revealing the role of a gut microbiota-dependent sclerotin-serotonin axis in disease (56). In the DBA/1 mouse model of spontaneous arthritis, levofloxacin treatment reduced pro-inflammatory bacteria like Prevotellaceae while increasing anti-inflammatory Muribaculaceae. This led to reduced inflammation in the gut, peripheral joints, and spine, alongside downregulation of SpA-associated cytokines and signaling pathways (57). Clinically, disease-modifying antirheumatic drugs (DMARDs) exert regulatory effects on gut microbiota. Tumor necrosis factor inhibitors (TNFi) not only effectively alleviate AS symptoms but also partially restore gut microbiota balance in patients, shifting microbial composition and functional profiles toward healthy controls—particularly by increasing SCFA-producing bacteria abundance (51, 58). Similarly, the JAK inhibitor tofacitinib, when treating psoriatic arthritis (PsA) and AS, has been observed to alter gut bacterial lineages, promoting the restoration of normal levels of genera such as *Bacteroides* and Coprobacter, which correlates with clinical improvement (59, 60). These findings suggest that modulating the gut microbiota may represent a novel adjunctive therapeutic strategy for SpA. Microbiome-targeted therapies, such as probiotics/prebiotics, have been proposed as adjunctive treatments for SpA, aiming to prevent or treat disease by regulating the gut microbiota and/or intestinal barrier function (61). However, more rigorously designed longitudinal studies and clinical trials are still needed to validate the long-term efficacy and safety of these interventions.

### 4.2 Role of the skin microbiome in psoriasis and psoriatic arthritis

As the body’s largest organ, the skin harbors a complex microbial community—the skin microbiome—on its surface. In psoriasis, the microbiome composition at affected sites differs significantly from non-affected areas and healthy skin. Although the literature primarily focuses on the gut microbiome, the universal principles of microbial-immune interactions revealed therein aid in understanding the skin microenvironment. Dysbiosis of the skin microbiome, such as increased colonization by *Staphylococcus aureus* and alterations in the balance of resident flora like Cutibacterium acnes, is thought to potentially exacerbate or perpetuate inflammatory skin states. Indeed, studies of the skin microbiome have demonstrated distinct microbial signatures in lesional and non-lesional skin of patients with psoriasis and psoriatic arthritis compared to healthy individuals (62, 63). These microbes activate pattern recognition receptors (e.g., Toll-like receptors) on keratinocytes and skin-resident immune cells (such as Langerhans cells) through their associated molecular patterns, triggering the release of pro-inflammatory cytokines. This process further recruits and activates additional immune cells, forming an inflammatory cycle (64). Although specific details of microbiome alterations in psoriatic lesions remain understudied in the literature, the core mechanism—where the microbiome influences adaptive immune responses via innate immunity—remains consistent. Disruption of the skin microbiome may compromise local immune tolerance and promote upregulation of pathogenic immune pathways like the IL-23/IL-17 axis, aligning closely with key pathophysiological mechanisms of psoriasis.

Psoriatic arthritis (PsA) represents a joint manifestation of psoriasis. While the gut-joint axis linking intestinal and joint pathologies has received considerable attention in its pathogenesis, the potential skin-joint axis warrants equal exploration. The emergence of joint symptoms in PsA patients may be associated with specific alterations in the skin and gut microbiome. Hypotheses suggest that chronic inflammation and dysbiosis in the skin or gut may activate local immune cells, which subsequently migrate to the joints and trigger arthritis. For example, Th17 cell expansion driven by gut dysbiosis and the migration of immune cells to the joints are considered key mechanisms in SpA pathogenesis (65). Although existing literature does not directly provide specific skin microbiome features predictive of progression from psoriasis to PsA, the paradigm of the microbiome influencing distal joints via systemic immunity is well-established. Future studies require longitudinal tracking of skin and gut microbiome dynamics in psoriasis patients to determine whether microbial biomarkers predictive of PsA onset exist. A small case series in PsA patients reported that tofacitinib therapy, while improving joint inflammation, also significantly altered gut microbiota composition, favoring bacterial strains considered beneficial for immunomodulation (59). This indirectly suggests bidirectional interactions between joints and the microbiome (including potentially affected skin microbiota), where therapeutic interventions can reshape microbial ecology while regulating systemic immunity.

**Figure 3:**
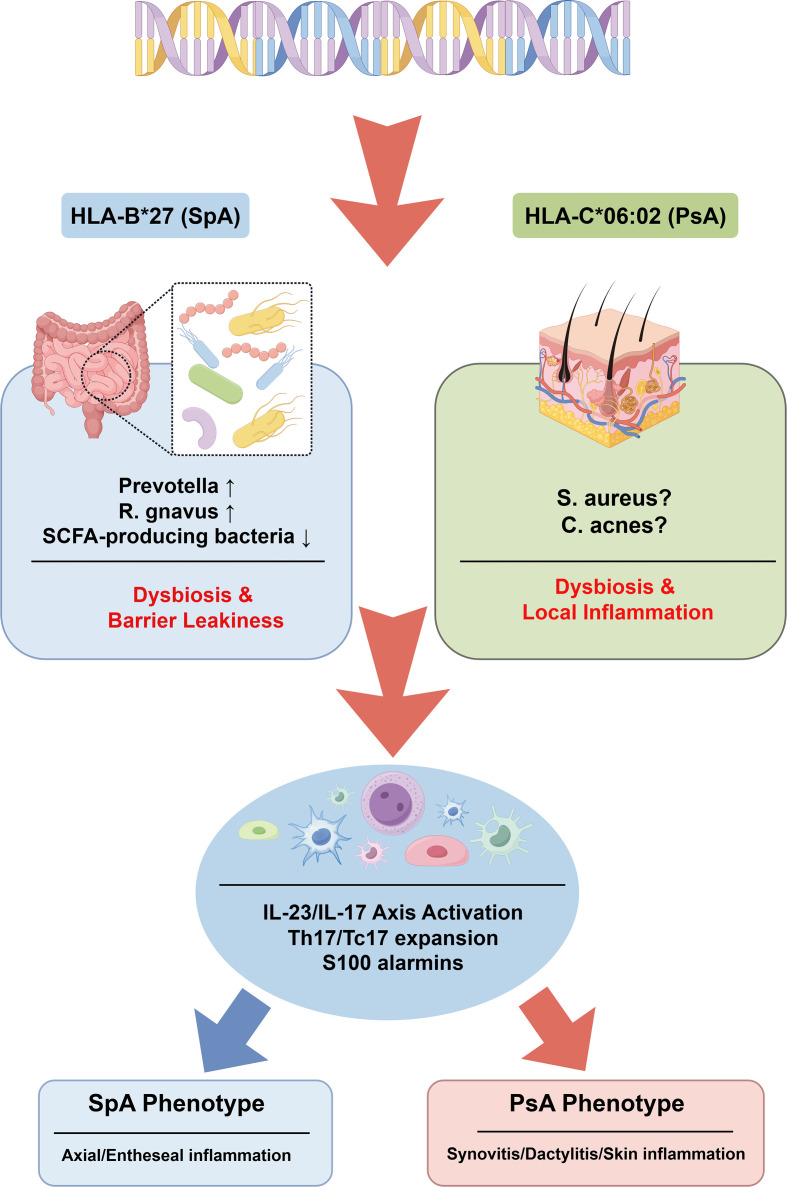
The dynamic interplay of host genetics and microbiome in shaping immunity in SpA and PsA. This schematic illustrates how genetic predisposition shapes site-specific microbiome alterations, which in turn drive distinct immunological pathways and clinical phenotypes. At the top, genetic risk variants—*HLA-B*27* (predominantly associated with SpA) and *HLA-C*06:02* (predominantly associated with PsA)—exert differential effects on the microbiome. In genetically susceptible individuals (*HLA-B*27* context, left pathway), the gut microbiome exhibits dysbiosis characterized by enrichment of Prevotella and *Ruminococcus gnavus*, depletion of SCFA-producing bacteria, and impaired intestinal barrier function. In the *HLA-C*06:02* context (right pathway), the skin microbiome shows dysbiosis involving potential pathobionts such as *Staphylococcus aureus* and altered commensals like Cutibacterium acnes, contributing to local epithelial inflammation. These site-specific microbial perturbations converge on a shared immune hub, driving systemic activation of the IL-23/IL-17 axis, expansion of Th17 and Tc17 cells, and release of alarmins (S100 proteins). From this hub, immune effectors diverge to target tissues, producing the characteristic phenotypes: axial and entheseal inflammation in SpA, and peripheral synovitis, dactylitis, and psoriatic skin lesions in PsA. This model highlights how gene-microbiome interactions at different body sites orchestrate the clinical diversity within the spondyloarthritis concept family. SCFAs, short-chain fatty acids; IL, interleukin; Th17, T helper 17; Tc17, IL-17-producing CD8^+^ T cell. Figure drawn by www.figdraw.com.

The core mechanism of microbiome involvement in psoriasis and PsA pathogenesis lies in its continuous dialogue with the host immune system. Skin and gut microbes persistently stimulate the host’s pattern recognition receptors (PRRs), particularly Toll-like receptors (TLRs), through their components (e.g., peptidoglycans, lipopolysaccharides) or metabolites. Activation of TLRs triggers downstream signaling pathways, including myeloid differentiation primary-response protein 88 (MyD88), leading to the activation of transcription factors such as nuclear factor κB (NF-κB) and activator protein 1 (AP-1). This, in turn, promotes the synthesis and release of proinflammatory cytokines, including TNF-α, IL-1β, IL-6, and IL-23 (64). IL-23 is a key upstream cytokine produced by activated dendritic cells and macrophages, driving the proliferation and stabilization of helper T cells 17 (Th17). Activated Th17 cells then massively secrete effector cytokines including IL-17A, IL-17F, and IL-22. In psoriasis and PsA, the IL-23/IL-17 axis is hyperactivated. IL-17 strongly stimulates keratinocyte proliferation, promotes neutrophil recruitment, and induces other proinflammatory mediators, leading to psoriasiform skin lesions and synovial inflammation (66). The microbiome is a potential initiator and regulator of this axis. For example, gut dysbiosis may disrupt immune tolerance to intestinal commensals by affecting antigen presentation, molecular mimicry, or direct superantigen exposure, leading to abnormal amplification of Th17 responses against self-antigens or cross-reactive microbial antigens (67). These abnormally activated immune cells may subsequently migrate via the bloodstream to the skin and joints, triggering or exacerbating inflammation. Consequently, targeting the microbiome or its downstream immune pathways—such as through IL-23 or IL-17 inhibitors—offers novel therapeutic approaches for psoriasis and PsA. **Figure 3** encapsulates the gene–microbiome–immune axis discussed in Section 4, showing how genetic background shapes site−specific microbial alterations that converge on systemic Th17/Tc17 activation and then diverge again to produce axial or peripheral disease. Complementing this visual model, **Table 3** consolidates the specific microbial signatures underlying these pathways. Together, these integrated views set the stage for the multi−omics integration strategies explored in Section 5.

**Table 3:** Gut and skin microbiome alterations in SpA and PsA.

Microbial feature	Finding in SpA/AS	Finding in PsA/PsO	Association with disease	References
Overall gut microbial diversity	Significantly reduced; similar to IBD	Characteristic alterations including reduced diversity	Dysbiosis characterizes disease state	(42, 49, 50)
Prevotella spp.	Enriched in AS gut	Not specifically reported	May promote pro-inflammatory state	(43)
Bacteroides	Reduced in AS gut	Restored by tofacitinib treatment	Loss of beneficial commensals	(43, 59, 60)
Proteobacteria/Enterobacteriaceae	Increased in AS gut (Han Chinese)	Not specifically reported	Potentially harmful bacteria enriched	(44)
Firmicutes/Actinobacteria	Reduced in AS gut	Not specifically reported	Beneficial bacteria depleted	(44)
Ruminococcus gnavus	Increased abundance in SpA; distinguishes patients from controls	Not specifically reported	Correlates with disease activity	(46)
Coprobacter spp.	Positively correlates with BASDAI and ASDAS-CRP; restored by tofacitinib	Restored by tofacitinib treatment	Potential disease activity biomarker	(47, 59, 60)
SCFA-producing bacteria (Megamonas, Lachnoclostridium, F. prausnitzii)	Significantly reduced in AS; negatively correlated with disease severity	Not specifically reported	Reduced anti-inflammatory metabolites; Th17/Treg imbalance	(51, 53)
Staphylococcus aureus	Not specifically reported	Increased colonization in psoriatic skin	May exacerbate skin inflammation via TLR activation	(62–64)
Cutibacterium acnes	Not specifically reported	Altered balance in psoriatic skin	Commensal dysbiosis contributes to inflammatory state	(62–64)
Tryptophan metabolites (IAA, I3Ald)	Increased in axSpA colon biopsies	Not specifically reported	Microbial genes for production enriched in axSpA	(52)
HLA-B27* effect	Shapes gut microbiota composition even in healthy siblings; drives pro-inflammatory state in mice	Not applicable	Genetic background influences microbiome	(46, 48)

This table consolidates evidence from metagenomic and metabolomic studies in human cohorts and animal models, demonstrating the characteristic dysbiosis patterns in gut and skin microbiomes and their association with disease activity and immunomodulation.

AS, ankylosing spondylitis; ASDAS-CRP, Ankylosing Spondylitis Disease Activity Score-C-reactive protein; axSpA, axial spondyloarthritis; BASDAI, Bath Ankylosing Spondylitis Disease Activity Index; HLA, human leukocyte antigen; IAA, indole-3-acetic acid; I3Ald, indole-3-acetaldehyde; IBD, inflammatory bowel disease; PsA, psoriatic arthritis; PsO, psoriasis; SCFA, short-chain fatty acid; SpA, spondyloarthritis; Th, T helper; TLR, Toll-like receptor; Treg, regulatory T cell.

## 5 Multi-omics data integration strategies and computational biology approaches

**Figure 4:**
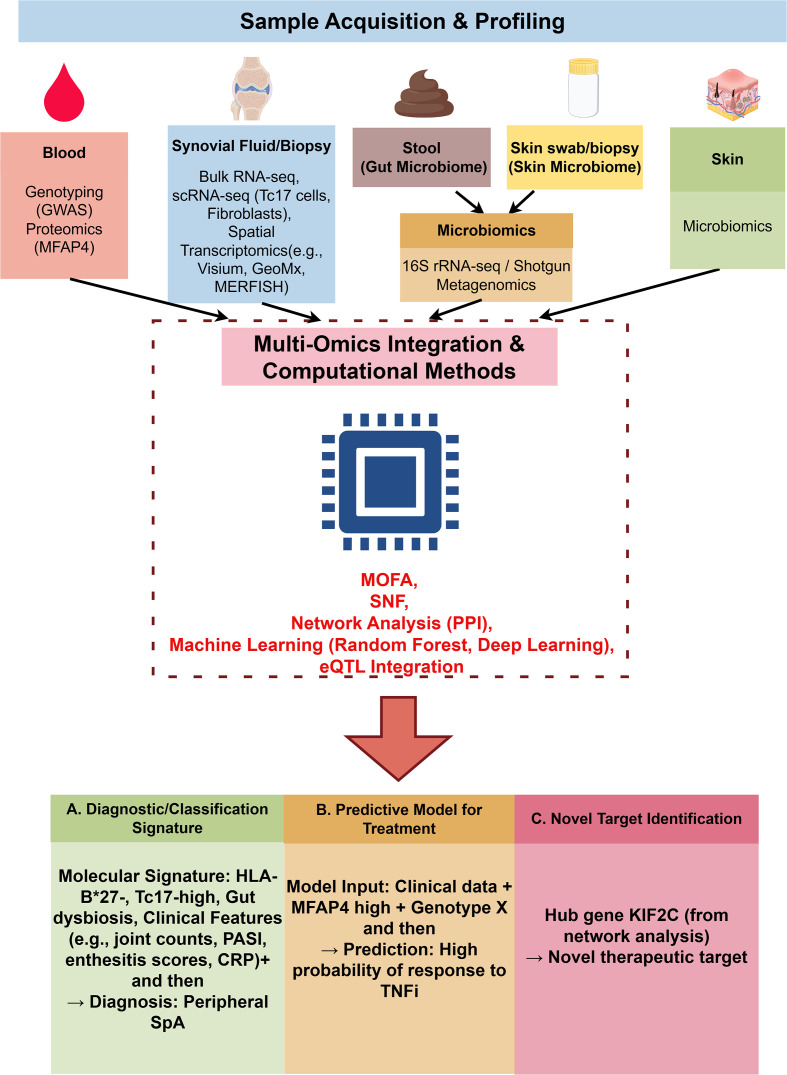
A multi-omics workflow for biomarker discovery and clinical implementation in SpA and PsA. This flowchart delineates the translational pipeline from biosample acquisition to clinical application. Step 1 (top) illustrates the collection of diverse biospecimens—blood, synovial fluid/tissue, stool (gut microbiome), and skin swab/biopsy (skin microbiome)—and their profiling using complementary omics technologies: genomics (GWAS, PRS), proteomics (e.g., MFAP4, S100 proteins), transcriptomics (bulk RNA-seq, scRNA-seq identifying key cell types such as Tc17 cells and activated fibroblasts, and spatial transcriptomics [e.g., Visium, GeoMx, MERFISH]), and microbiomics (16S rRNA sequencing/metagenomics). Step 2 (middle) depicts the computational integration hub, where multi-omics data are fused using advanced bioinformatics approaches, including multi-omics factor analysis (MOFA), similarity network fusion (SNF), protein-protein interaction (PPI) network analysis, and machine learning algorithms (random forest, deep learning), with integration of expression quantitative trait locus (eQTL) data to link genetic variation to functional consequences. Step 3 (bottom) presents three translational outputs that incorporate both molecular signatures and clinical features (e.g., CRP, PASI, joint counts, enthesitis scores): **(A)** diagnostic/classification signatures enabling molecular subtyping (e.g., “gut-driven” peripheral SpA vs. “skin-driven” PsA); **(B)** predictive models forecasting treatment response (e.g., high baseline MFAP4 predicting favorable response to TNFi therapy); and **(C)** identification of novel hub genes and pathways as candidate therapeutic targets. This integrated workflow exemplifies the path toward precision rheumatology. Abbreviations: GWAS, genome-wide association study; PRS, polygenic risk score; MFAP4, microfibrillar-associated protein 4; scRNA-seq, single-cell RNA sequencing; TNFi, tumor necrosis factor inhibitor; CRP, C-reactive protein; PASI, Psoriasis Area and Severity Index. Figure drawn by www.figdraw.com.

To bridge the gap between omics technologies and patient care, **Figure 4** presents an integrated translational pipeline. It emphasizes how computational methods (e.g., MOFA, SNF, deep learning) convert raw molecular data into tools for early diagnosis, treatment response prediction, and novel target identification.

### 5.1 Challenges and methodologies in multi-omics data integration

Integrating heterogeneous multi-omics data to construct a coherent biological model of SpA and PsA presents formidable challenges. These stem from fundamental differences in data types (e.g., discrete SNPs vs. continuous protein measurements), technical variations across platforms (batch effects), and the asynchronous dynamics of molecular changes over time (68–70). Furthermore, the high dimensionality and frequent incompleteness of these datasets complicate the separation of true biological signals from noise and technical artifacts (71, 72). This challenge is particularly acute in skin microbiome studies, where the inherently low microbial biomass necessitates rigorous experimental controls and specialized computational methods to distinguish genuine dysbiosis from environmental contamination.

To address these challenges, computational strategies have been developed to extract complementary information from these diverse datasets. One class of methods aims to identify shared drivers of variation across omics layers. For example, Multi-Omics Factor Analysis (MOFA) uses dimensionality reduction to project data onto a low-dimensional space, revealing common factors that explain variation in multiple datasets (73). MOFA has been successfully applied to integrate multi-omics data in inflammatory bowel disease (IBD), disentangling sources of variation driven by genetics, gut microbiome composition, and metabolomics to reveal latent factors linked to disease activity and treatment response (74, 75). Another strategy focuses on capturing sample similarities. Similarity Network Fusion (SNF) constructs individual networks for each omic dataset and fuses them into a robust consensus network for disease subtyping (76). SNF has demonstrated utility in subtyping complex autoimmune diseases by fusing genomic, transcriptomic, and clinical data to identify patient clusters with distinct molecular profiles and differential responses to therapy—a framework with direct translational relevance for the heterogeneity observed in SpA and PsA (77). More recently, deep learning models have been employed to model complex, non-linear interactions between modalities (78, 79).

After obtaining the integrated list of molecular features, functional interpretation within a biological context is required. Pathway enrichment analysis serves as a foundational tool. By mapping differentially expressed genes, proteins, or metabolites to known pathway databases such as KEGG or Reactome, biologically significant pathways significantly dysregulated in disease—such as inflammation or immune-related pathways—can be identified (70). However, deeper insights require network biology approaches. For instance, constructing protein-protein interaction (PPI) networks reveals physical interactions among differentially expressed proteins and identifies key hub proteins within the network, which may serve as potential therapeutic targets (80). Gene co-expression network analysis identifies gene modules that change synergistically under disease conditions, often associated with specific cellular functions or pathways (81). More advanced integrative approaches like mBONITA leverage Boolean networks to integrate quantified multi-molecular-level omics datasets. By combining prior knowledge networks with topology-based pathway analysis, it identifies genes consistently regulated across multiple omics measurements, thereby revealing cross-omics coordinated regulatory mechanisms (70). Through these approaches, researchers can transform vast multi-omics data into interpretable biological hypotheses. For instance, they may identify specific immune-inflammatory pathways in SpA/PsA that are simultaneously influenced by genetic variation, dysregulated at transcriptional and translational levels, and modulated by gut microbial metabolites. The comparative overview in **Table 4** provides researchers with a framework for selecting appropriate integration strategies based on specific research questions. Section 5.2 demonstrates the translational power of these approaches through case studies and their application to precision medicine in SpA and PsA.

**Table 4:** Computational methods for multi-omics data integration in SpA and PsA research.

Method	Category	Principle	Application in SpA/PsA	Advantages	Limitations	References
MOFA (Multi-Omics Factor Analysis)	Dimensionality reduction	Projects multi-omics data onto low-dimensional space; identifies shared factors explaining variation across datasets	Identifying common drivers of variation across genomic, transcriptomic, and proteomic layers; applied in IBD to reveal factors linked to disease activity	Handles multiple data types; reveals latent factors; interpretable	May miss rare variants; requires careful parameter tuning	(73–75)
SNF (Similarity Network Fusion)	Network-based	Constructs sample similarity networks for each omic dataset; fuses into consensus network	Disease subtyping based on integrated molecular profiles; demonstrated in complex diseases for identifying patient clusters with distinct molecular profiles	Captures multi-scale structure; robust to noise; handles heterogeneous data	Computationally intensive; network construction parameters critical	(76, 77)
PPI network analysis	Network biology	Maps physical interactions among differentially expressed proteins; identifies hub proteins	Identifying key hub proteins as potential therapeutic targets	Biologically interpretable; leverages prior knowledge	Dependent on existing interaction databases; may miss novel interactions	(80)
Gene co-expression network analysis	Network biology	Identifies gene modules that change synergistically under disease conditions	Identifying modules associated with specific cellular functions or pathways	Reveals coordinated regulation; module-based interpretation	Correlation-based; does not imply causality	(81)
mBONITA	Boolean network integration	Integrates quantified multi-molecular-level omics datasets; combines prior knowledge networks with topology-based pathway analysis	Identifying genes consistently regulated across multiple omics measurements; revealing cross-omics coordinated regulatory mechanisms	Integrates multiple molecular levels; leverages prior knowledge; identifies consistent regulators	Requires quantified multi-omics data; Boolean assumptions may oversimplify	(70)
Random forests	Machine learning (traditional)	Ensemble learning method combining multiple decision trees	Predicting treatment response to biologics or JAK inhibitors from integrated omics features	Handles high-dimensional data; provides feature importance; interpretable	May overfit without careful tuning; limited in capturing complex non-linear relationships	(82)
Support vector machines	Machine learning (traditional)	Finds optimal hyperplane separating classes in high-dimensional space	Classification for differential diagnosis; prediction of treatment response	Effective in high-dimensional spaces; robust to overfitting	Kernel selection critical; less interpretable than random forests	(82)
Deep learning	Deep learning	Neural networks for modeling complex non-linear relationships	Predicting treatment response; integrating heterogeneous data types (genomics, transcriptomics, clinical parameters)	Captures complex non-linear interactions; handles heterogeneous data types	“Black box” nature; requires large sample sizes; computationally intensive	(78, 79, 82)

This table consolidates evidence from bioinformatics and machine learning applications, demonstrating the principles, advantages, and limitations of various integration methodologies for transforming multi-omics data into biologically and clinically meaningful insights.

MOFA, Multi-Omics Factor Analysis; PPI, protein-protein interaction; PsA, psoriatic arthritis; SNF, Similarity Network Fusion; SpA, spondyloarthritis.

### 5.2 Building predictive models and identifying core pathways

Integrating multi-omics features using machine learning algorithms provides powerful tools for precision medicine in SpA/PsA. Traditional algorithms like random forests and support vector machines are widely applied due to their interpretability and ability to handle high-dimensional data. In the specific context of SpA and PsA, machine learning models are beginning to show promise in predicting treatment response. A systematic review of artificial intelligence applications in this field found that models integrating clinical and multi-omics data could predict response to biologics or JAK inhibitors with moderate to high accuracy (area under the curve [AUC] ranging from 0.63 to 0.92) (82). These models often highlight key predictive features, such as specific genetic variants (e.g., in TNF or IL17 pathway genes), baseline proteomic markers (e.g., elevated S100A8/A9 or MMP-3), or distinct gut microbial profiles. Deep learning models, which excel at capturing complex nonlinear relationships, are also being explored for their ability to integrate heterogeneous data types—including genomics, transcriptomics, and clinical parameters—to further improve predictive performance (78, 79). These models integrate multidimensional features from genomics, epigenomics, transcriptomics, proteomics, and metabolomics to construct classification or regression models for SpA/PsA. Applications include differential diagnosis (e.g., distinguishing from other inflammatory arthritides), disease activity grading (e.g., predicting BASDAI or DAPSA scores), and treatment response prediction (e.g., response to TNF inhibitors or IL-17 inhibitors). Key challenges in model development include feature selection, addressing data imbalance, and preventing overfitting through cross-validation, with the ultimate goal of creating clinically applicable, robust predictive tools.

Case studies clearly demonstrate how integrated analysis identifies core regulatory pathways and key hub molecules beyond single-omics approaches. In the context of inflammatory diseases, integrative multi-omics analyses have successfully identified core regulatory pathways that would be difficult to discern from a single omics layer. For example, in SpA and PsA, a potential core pathway involves immune-inflammatory cascades that are simultaneously influenced by genetic variants such as *HLA-B*27* (genome), exhibit upregulation of downstream inflammatory mediators such as IL-17 and TNF-α at both mRNA and protein levels (transcriptome/proteome), and are further modulated by the activity of gut microbiota-derived metabolites such as short-chain fatty acids (microbiome) (83). Identifying such cross-omics hub molecules and pathways not only deepens our understanding of disease pathogenesis but also provides a direct rationale for developing novel biomarkers and targeted therapeutic strategies.

## 6 Application potential of multi-omics biomarkers in clinical diagnosis and differential diagnosis

### 6.1 Early diagnosis and disease classification

Early diagnosis of spondyloarthritis (SpA) and psoriatic arthritis (PsA) presents significant challenges, particularly in the early stages of non-radiographic axial SpA (nr-axSpA) and PsA. The diagnostic dilemma is particularly pronounced for nr-axSpA, where the absence of definitive radiographic evidence of sacroiliitis necessitates reliance on clinical features, *HLA-B*27* status, and magnetic resonance imaging (MRI) (84). The Association for Spondyloarthritis (ASAS) recently proposed a consensus definition for early axSpA, defined as axial symptoms lasting ≤2 years, providing a framework for standardized research. However, diagnostic delays remain a significant clinical challenge. While historical data reported delays averaging up to 8.5 years (85, 86), more contemporary registry-based studies indicate that this interval is shortening in some healthcare settings, though substantial geographical and healthcare-system variability persists (87–89). This variability underscores the need for universally applicable, objective biomarkers to expedite diagnosis regardless of healthcare context. Diagnosing early PsA is equally challenging due to its variable symptoms and frequent overlap with psoriatic dermatosis (PsO) or other inflammatory arthritis. Over 80% of PsA cases have a history of psoriasis prior to arthritis onset, yet fewer than 30% of PsO patients progress to PsA, making high-risk identification among PsO populations highly difficult (90). Developing objective molecular biomarkers is urgently needed to overcome these clinical challenges, enable early intervention, and improve prognosis.

Multi-omics-based signature combinations demonstrate potential efficacy in distinguishing early SpA/PsA from other diseases. For differentiating SpA from other inflammatory arthritides, serum galectin-1 (Galectin-1) levels have been shown to be significantly lower in SpA patients compared to rheumatoid arthritis (RA) patients, suggesting its potential as a diagnostic biomarker (91). For distinguishing PsA from uncomplicated psoriasis, studies indicate that specific clinical features in PsO patients (e.g., nail involvement, untreated plaque psoriasis) and plasma microRNA levels (e.g., hs-miR-210-3p) correlate with increased PsA risk (92, 93). Furthermore, synovial fluid analysis in early inflammatory arthritis revealed significantly higher frequencies of IL-17A+ CD8+ T cells (Tc17) in SpA patients compared to seronegative undifferentiated arthritis patients, suggesting specific activation of the Tc17 pathway in early SpA (29). These findings suggest that multi-omics detection panels integrating specific gene expression profiles (e.g., Tc17-associated genes), serum protein combinations (e.g., Galectin-1), and microbial biomarkers hold promise for improving the accuracy of early differential diagnosis.

Integrating these biomarkers into existing classification criteria has the potential to significantly enhance diagnostic sensitivity and specificity. Current ASAS classification criteria and the Classification of Psoriatic Arthritis (CASPAR) are widely used in clinical practice but are sometimes misapplied as diagnostic tools, leading to misdiagnosis or missed diagnosis (94). For example, ASAS criteria rely on clinical features, *HLA-B*27*, and MRI, yet exhibit limited specificity (95). Incorporating multiple sets of biomarkers as supplements to these criteria could provide a more objective, quantifiable dimension. For instance, quantifying bone marrow edema (BMO) on sacroiliac joint MRI in patients with suspected axSpA has been shown to enhance predictive capability, particularly in *HLA-B*27*-negative patients (96). For PsA, integrating imaging features detected by ultrasound—such as enthesitis and synovitis—with serum biomarkers (e.g., specific cytokine profiles) can optimize the application of CASPAR criteria, particularly in cases lacking typical psoriatic skin lesions (97). This integration holds promise for reducing diagnostic delays, enabling more precise disease classification, and ultimately delivering timelier, personalized treatment to patients.

### 6.2 Molecular features distinguishing SpA from PsA

At the genomic, transcriptomic, proteomic, and microbiome levels, several candidate biomarker combinations have been identified that can relatively specifically distinguish SpA (particularly ankylosing spondylitis, AS) from PsA. Genomically, *HLA-B*27* exhibits a strong association with SpA, especially AS, whereas this link is weaker in PsA; conversely, PsA shows a closer association with alleles such as *HLA-C*06:02* (90). Transcriptomic analysis reveals significantly elevated frequencies of Tc17 cells (IL-17A-producing CD8+ T cells) in synovial fluid from early-stage SpA patients, whereas CD8+ IFNγ+ T cells dominate in RA patients. This provides clues for distinguishing SpA (including PsA) from RA, though more refined transcriptomic profiles may be needed to differentiate AS from PsA (29). Proteomics studies reveal lower serum galectin-1 levels in SpA and higher levels in RA, though its value in differentiating AS from PsA requires further clarification (91). Regarding the microbiome, SpA is closely associated with gut dysbiosis, particularly alterations linked to the “mucosal origin” hypothesis (98). In contrast, PsA may exhibit a more complex interplay involving both gut and, distinctively, skin microbiome dysbiosis, the latter of which is discussed in Section 4.2. Integrating these multi-omics features—such as *HLA-B*27* status, specific Tc17 cell frequencies, serum protein markers (e.g., Galectin-1, S100 proteins), and gut or skin microbiota characteristics—may yield a biomarker combination to differentiate AS from PsA.

These differential features carry significant immunological implications. SpA, particularly AS, exhibits stronger *HLA-B*27* association and gut dysbiosis, supporting the “mucosal origin” hypothesis. This hypothesis posits that immune dysregulation and compromised barrier function in the intestinal mucosa lead to bacterial antigen translocation, triggering abnormal immune responses against axial joints and enthesitis sites in genetically susceptible individuals (e.g., *HLA-B*27*-positive) (99, 100). Experimental models also demonstrate that neutrophils play a crucial role in early enthesitis by producing alarmins like S100A8/A9, consistent with the inflammatory mechanisms of the mucosal-articular axis (100). In contrast, the immunological characteristics of PsA may be more oriented toward stronger cytotoxic signaling and skin microbiota-immune interactions. PsA is closely associated with psoriatic skin lesions, where skin barrier disruption and local microbiome alterations may drive IL-23/IL-17 axis-mediated immune responses affecting not only the skin but also joints and entheses (90). Furthermore, the expansion of Tc17 cells in peripheral blood of PsA patients underscores the role of cytotoxic T cells in disease pathogenesis (29). Thus, the immunopathology of SpA may emphasize interactions between the mucosal immune system (particularly the gut) and innate immunity (e.g., neutrophils), whereas PsA may focus more on the skin-joint axis and the dominant role of Th17/Tc17 pathways in adaptive immunity. Understanding these distinctions is crucial for developing precision therapeutic strategies targeting specific immune pathways.

## 7 Translational prospects for guiding treatment decisions and prognosis assessment

### 7.1 Predicting treatment response and precision subtyping

Multi-omics research demonstrates significant potential in predicting treatment responses to drugs with different mechanisms of action in patients with spondyloarthritis (SpA) and psoriatic arthritis (PsA). Despite successful targeted therapies, the absence of predictive biomarkers often leads to trial-and-error approaches in clinical practice, resulting in inconsistent outcomes (13). Artificial intelligence (AI) technologies, particularly supervised machine learning (e.g., random forests, support vector machines) and deep learning, have been applied to integrate electronic health records, clinical biomarkers, genetic, and proteomic data to predict patient responses to biologics and JAK inhibitors. These models achieve prediction accuracies ranging from 60% to 70%, with AUC values between 0.63 and 0.92 (82). For instance, in predicting the efficacy of secukinumab (an IL-17 inhibitor) in PsA patients with axial manifestations, studies identified nail dystrophy as a predictor of treatment response (101). Furthermore, patients with chronic inflammatory diseases (including PsA and axial SpA) exhibiting elevated baseline serum microfibrillar-associated protein 4 (MFAP4) levels demonstrated a higher likelihood of achieving a positive clinical response after initiating biologic therapy (12). Smoking status has also been confirmed as a significant factor influencing treatment response, with smokers—particularly those with rheumatoid arthritis—showing markedly reduced response rates to biologics (102). These studies underscore the importance of leveraging multi-omics data to construct predictive models for more precise treatment allocation.

Molecular subtyping of SpA and PsA patients using baseline multi-omics features is key to achieving precision-matched targeted therapies. Single-cell RNA sequencing (scRNA-seq) studies have revealed immune cell heterogeneity in autoimmune arthritis, providing insights into disease mechanisms and variations in treatment responses (8). By integrating genomic, proteomic, and microbiome data, patients can be classified into subtypes with distinct molecular signatures, such as “Th17-dominant,” “type I interferon-dominant,” or “gut-driven.” The Systemic Immune Inflammation Index (SII), a biomarker reflecting inflammatory burden and immune dysregulation, correlates with disease activity scores, musculoskeletal imaging findings, and treatment response in SpA and PsA, suggesting its utility in identifying distinct immune phenotypes (103). In PsA, ultrasound (US)-based assessment has been systematically applied for diagnosis and management, with practical algorithms proposed from diagnosis to treatment response evaluation. This facilitates stratified management based on specific patient presentations (104). The identification of these molecular and immunological subtypes enables matching the most effective targeted therapies to specific subtypes (e.g., IL-17 inhibitors for “Th17-dominant” subtypes), potentially improving treatment response rates (13).

Dynamic monitoring of multi-omics biomarker changes during treatment offers the feasibility of early identification of responders versus non-responders and timely adjustment of therapeutic regimens. Artificial intelligence models can integrate multi-source data during treatment to enable dynamic prediction (82). For example, in chronic inflammatory diseases, assessing clinical response and biomarker changes 14–16 weeks after treatment initiation can identify factors associated with sustained efficacy (12). In PsA management, ultrasound serves as an objective assessment tool to guide treatment adjustments for patients with suboptimal clinical response (104). Furthermore, model-informed drug development approaches have successfully bridged subcutaneous to intravenous administration of secukinumab through population pharmacokinetic analysis and exposure-response analysis, predicting efficacy and safety. This demonstrates the potential for optimizing treatment strategies through pharmacokinetic dynamic monitoring (105). Although this field remains in its early stages and faces challenges such as methodological heterogeneity, data integration, and external validation, achieving personalized treatment adjustments through dynamic multi-omics monitoring undoubtedly represents a key future direction for rheumatology clinical practice (13, 82).

### 7.2 Identification of novel therapeutic targets and drug development

Core pathogenic pathways and key regulatory molecules identified through multi-omics integration analyses provide a rich resource for screening novel drug targets in SpA and PsA. Single-cell RNA sequencing studies have revealed heterogeneity in synovial fibroblasts and immune cell subsets (e.g., peripheral helper T cells and clonally expanded CD8+ T cells). These findings not only elucidate disease mechanisms but also point to potential therapeutic targets (8). For instance, in SpA and PsA, bone remodeling is driven by inflammatory cytokines like tumor necrosis factor-α (TNFα) and interleukin-17A (IL-17A), which promote osteoclastogenesis via the RANKL pathway and suppress osteoblast-mediated bone formation through WNT/β-catenin signaling (106). Multi-omics analysis aids in identifying key regulatory molecules within these pathways, such as novel cytokines, chemokines, metabolic enzymes, or microbial products. Expert consensus indicates that in the SpA field, further understanding is needed regarding the role of interleukin-23 (IL-23) in pathogenesis, as well as the genetic relationship between IL-23 receptor polymorphisms and other associated systemic inflammatory diseases (e.g., inflammatory bowel disease), which may represent novel therapeutic targets (107). By integrating genomic, transcriptomic, proteomic, and metabolomic data with bioinformatics modeling, core molecules driving specific disease subtypes can be systematically identified, laying the foundation for developing novel targeted therapeutics (108).

Microbiome-based intervention strategies offer novel adjunctive treatment possibilities for SpA and PsA. Gut microbiota composition constitutes a key component of the multi-omics profile in SpA and PsA, with specific dysbiosis potentially correlating to disease subtypes (e.g., “gut-driven” variants). Microbiome-targeted interventions include designing phages or antibiotics against specific pathogenic bacteria to eliminate harmful flora. More importantly, employing specific probiotics, prebiotics, or postbiotics can modulate microbial function, restore gut microecological balance, and thereby indirectly regulate systemic immune responses. More direct microbiome-modulating therapies, such as fecal microbiota transplantation (FMT), are in their infancy for rheumatic diseases. While evidence in SpA and PsA remains limited to case reports and small heterogeneous series, early results suggest a potential role in modulating disease activity, highlighting the need for rigorously designed clinical trials (109, 110). While the current literature does not directly detail clinical trials of microbiome interventions for SpA/PsA, the framework of multi-omics research supports this direction. For instance, in chronic inflammatory diseases like inflammatory bowel disease (IBD), microbiome research has been applied to predict treatment responses and develop novel therapies (111). Applying similar strategies to SpA and PsA—by modulating immune pathways such as Th17 cell differentiation through gut microbiota regulation—holds promise as an effective adjunct to existing targeted therapies. The expert meeting also emphasized the need to continue developing therapies and strategies for refractory diseases, where microbiome-based interventions may represent a promising avenue (112). Future research could therefore explore how to leverage multi-omics data to identify key disease-associated microbial signatures and design precision microbiome-targeted interventions accordingly. While **Table 5** illustrates the substantial translational potential of multi-omics biomarkers, the path from discovery to clinical implementation is fraught with obstacles. The following section critically examines the technical hurdles, validation requirements, and ethical imperatives that will shape the future of this field.

**Table 5:** Clinical applications and translational potential of multi-omics biomarkers in SpA and PsA.

Application area	Biomarker/Signature	Finding	Clinical utility	References
Early diagnosis (nr-axSpA)	*HLA-B*27*+ MRI bone marrow edema quantification	BMO quantification enhances predictive capability, especially in *HLA-B*27*-negative patients	Improved early detection; may reduce diagnostic delays	(84–86, 96)
Early diagnosis (PsA)	Clinical features (nail involvement, untreated plaque psoriasis) + plasma microRNAs (hs-miR-210-3p)	Correlate with increased PsA risk among PsO patients	Identify high-risk PsO patients for monitoring and early intervention	(92, 93)
Differential diagnosis (SpA vs. RA)	Serum galectin-1	Significantly lower in SpA compared to RA	Potential diagnostic biomarker to distinguish SpA from RA	(91)
Differential diagnosis (SpA vs. undifferentiated arthritis)	Synovial fluid Tc17 cell frequency	Significantly higher in early SpA	Identifies specific activation of Tc17 pathway in early SpA	(29)
Differential diagnosis (pSpA vs. PsA sine psoriasis)	*HLA-B*27*, *HLA-C*06:02*, clinical features (enthesitis, dactylitis, DIP involvement)	pSpA: male predominance, *HLA-B*27*^+^, enthesitis, large lower limb joints; PsA sine psoriasis: *HLA-C*06:02*^+^, dactylitis, hand DIP involvement	Enables more precise disease classification and appropriate treatment	(5)
Treatment response prediction (biologics)	Baseline serum MFAP4	Elevated levels correlate with favorable clinical response after biologic initiation	Identify patients likely to respond to biologics; reduce trial-and-error	(12)
Treatment response prediction (secukinumab)	Nail dystrophy	Predicts response to secukinumab in PsA patients with axial manifestations	Guide IL-17 inhibitor therapy selection	(101)
Treatment response prediction (biologics)	Smoking status	Smokers show reduced response rates to biologics	Modifiable factor; inform patient counseling	(102)
Treatment response prediction (TNFi, IL-17i)	eQTL genotypes associated with key pathway genes	Predict pre-treatment expression levels of relevant proteins; establish genetic-protein-efficacy association	Enable pre-treatment identification of responders vs. non-responders	(31, 41)
Molecular subtyping	“Th17-dominant,” “type I interferon-dominant,” “gut-driven” subtypes	Identified through integrated genomic, proteomic, and microbiome data	Match targeted therapies (e.g., IL-17 inhibitors for Th17-dominant subtype)	(13, 82)
Disease activity monitoring	Systemic Immune Inflammation Index (SII)	Correlates with disease activity scores, imaging findings, and treatment response	Reflects inflammatory burden and immune dysregulation	(103)
Treatment response monitoring	Ultrasound assessment	Objective tool to guide treatment adjustments for suboptimal responders	Enable dynamic treatment optimization	(104)
Novel therapeutic targets	scRNA-seq-identified cell subsets (peripheral helper T cells, clonally expanded CD8^+^ T cells)	Reveal disease-relevant cell populations	Point to potential cell-targeted therapeutic strategies	(8)
Novel therapeutic targets	Bone remodeling pathway molecules (RANKL, WNT/β-catenin)	Driven by TNFα and IL-17A; regulate osteoclastogenesis and bone formation	Identify key regulatory molecules for therapeutic intervention	(106)
Microbiome-based interventions	Gut microbiota composition	Specific dysbiosis correlates with disease subtypes; modulated by TNFi and JAK inhibitors	Novel adjunctive treatment possibilities; probiotics/prebiotics/FMT as emerging strategies	(51, 58–61, 109–112)

This table consolidates evidence from clinical cohort studies and translational research, demonstrating the utility of multi-omics biomarkers across the spectrum of early diagnosis, differential diagnosis, treatment response prediction, and precision medicine strategies.

BMO, bone marrow edema; DIP, distal interphalangeal; eQTL, expression quantitative trait locus; HLA, human leukocyte antigen; IL, interleukin; MFAP4, microfibrillar-associated protein 4; MRI, magnetic resonance imaging; nr-axSpA, non-radiographic axial spondyloarthritis; PsA, psoriatic arthritis; PsO, psoriasis; pSpA, peripheral spondyloarthritis; RA, rheumatoid arthritis; scRNA-seq, single-cell RNA sequencing; SII, Systemic Immune Inflammation Index; SpA, spondyloarthritis; Tc17, IL-17-producing CD8+ T cell; Th, T helper; TNFi, tumor necrosis factor inhibitor.

## 8 Current challenges, future directions, and ethical considerations

### 8.1 Technical, validation, and standardization challenges

Current multi-omics research on spondyloarthritis and psoriatic arthritis faces significant technical and validation bottlenecks. Most studies employ cross-sectional designs with limited sample sizes and lack independent validation cohorts, severely restricting the reliability and generalizability of findings. For instance, a systematic review on AI-driven treatment response prediction noted that while multi-omics approaches show promise in predicting biologic response, methodological heterogeneity limits their generalizability (82). Therefore, advancing large-scale, longitudinal, multi-center cohort studies is crucial to capture disease dynamics and ensure biomarker robustness. The TOFA-PREDICT trial design serves as a positive example, planning to enroll 160 patients and develop predictive models using data from the first 80 patients, with the latter 80 used for validation, demonstrating a commitment to independent validation (113). Transforming high-dimensional multi-omics data from laboratory discoveries into clinically actionable, simplified biomarker panels presents another major challenge. This involves translating complex genomics and proteomics discoveries into stable, multi-marker detection methods based on PCR or immunoassays that can be routinely performed in clinical laboratories. For instance, an integrated multi-omics analysis identified seven plasma proteins associated with psoriatic arthritis risk, such as IL23R and ERAP2; however, translating these findings into standardized diagnostic kits still requires overcoming technical translation and standardization hurdles (114). Furthermore, data sharing and establishing public multi-omics databases are critical for advancing the field. The absence of unified analytical workflows and public data resources hinders the comparison and integration of disparate research findings. Establishing a cloud-based data management hub—similar to those used in vaccine research—for centralized management, quality control, and sharing of multi-omics data would significantly accelerate biomarker discovery and validation processes (115). Standardizing the entire workflow—from sample collection and data processing to analytical reporting—is fundamental to ensuring the reproducibility, comparability, and ultimate clinical translation of future multi-omics biomarker research.

### 8.2 Future research directions and clinical integration pathways

Future research should focus on identifying multi-omics signatures in high-risk individuals during the “preclinical stage” before disease onset to achieve true disease prevention and ultra-early intervention (116). For psoriatic arthritis, identifying high-risk subgroups among psoriasis patients who progress to arthritis is a current research priority, with multi-omics approaches poised to reveal key molecular events in this transition. Drawing from research approaches in latent tuberculosis infection, integrating immunological, transcriptomic, and metabolomic data to identify preclinical individuals at progression risk is equally applicable to the field of spondyloarthritis (117). Secondly, it is crucial to advocate for interventional clinical trials integrating multi-omics data. Such trials can directly evaluate whether biomarker-based stratified treatment strategies outperform standard therapies. The TOFA-PREDICT trial exemplifies this approach, integrating clinical, molecular, and imaging parameters to develop models predicting tofacitinib efficacy and directly comparing treatment responses (113). The subsequent predictive model developed from clinical data demonstrated potential for forecasting treatment responses based on baseline clinical features, with plans to further integrate imaging and multi-omics biomarkers to enhance predictive performance (118). Finally, successfully integrating multi-omics biomarkers into clinical pathways requires establishing multidisciplinary teams encompassing rheumatology, dermatology, bioinformatics, and computational biology experts (82). Concurrently, feasibility studies and health economic evaluations must be conducted to determine the cost-effectiveness of these novel assays and design rational clinical implementation pathways. For instance, in the field of targeted asthma therapy, expert consensus emphasizes the need to optimize implementation strategies and address barriers such as resource limitations and cost-effectiveness—insights that hold significant relevance for rheumatology (119). Only through such systematic research design and interdisciplinary collaboration can multi-omics scientific discoveries be translated into actionable clinical tools, ultimately realizing personalized medicine.

### 8.3 Ethics, privacy, and accessibility

Multi-omics research involves vast amounts of personal genetic, molecular, and microbiological data, raising critical ethical issues regarding privacy protection, data security, and informed consent. These highly sensitive data can have profound consequences for individuals if compromised. Therefore, studies must establish rigorous data security management frameworks, such as employing cloud storage and access control technologies, while obtaining comprehensive, dynamic informed consent during sample collection that clearly outlines the scope of data use and potential risks (115). Furthermore, the development of multi-omics biomarker detection may face high cost barriers, potentially leading to unequal distribution of healthcare resources and exacerbating existing health disparities. Ensuring equitable access to these advanced detection technologies presents a major challenge. This requires consideration at multiple levels: during the R&D phase, efforts should focus on developing cost-effective detection methods; At the policy level, health economic evaluations are needed to demonstrate long-term value and secure inclusion in health insurance reimbursement schemes. Implementation must prioritize accessibility in resource-constrained regions to prevent technology from benefiting only select populations (119). For instance, in cancer immunotherapy, efforts to identify predictive biomarkers for treatment response have underscored the importance of multidisciplinary alliances and data-sharing platforms to reduce costs and promote equity (120). Similarly, in rheumatology, advancing the discovery of psoriatic arthritis biomarkers requires international collaboration among patient organizations, clinicians, researchers, industry partners, and regulators, alongside securing public-private funding to ensure robust biomarkers gain broad market acceptance and adoption (116). In summary, while pursuing scientific advancement, it is imperative to proactively address ethical, privacy, and accessibility concerns to ensure multi-omics technologies benefit all patients in an ethical and equitable manner.

## 9 Conclusion

From a systems biology perspective, integrated multi-omics analyses have fundamentally reshaped our understanding of SpA and PsA. They reveal a landscape of profound commonalities, centered on pathways like the IL-23/Th17 axis, while simultaneously highlighting subtle but critical distinctions in genetic architecture and tissue-specific immune dysregulation that underpin their divergent clinical phenotypes.

The central goal of this field is to translate these multidimensional data into clinically actionable tools. This endeavor marks the transition of rheumatology from a paradigm largely reliant on clinical and radiographic assessment towards a new era of precision medicine, where decisions are guided by a patient’s unique molecular profile.

In summary, the integrated multi-omics perspective underscores that SpA and PsA, while united by a common pathogenic backbone centered on the IL-23/IL-17 axis and gut–joint immune crosstalk, diverge in significant ways. SpA (particularly AS) is characterized by a stronger genetic imprint of *HLA-B*27*, a more pronounced gut microbial dysbiosis signature, and a dominant mucosal-articular axis of inflammation. In contrast, PsA exhibits a more robust association with *HLA-C*06:02*, a distinct transcriptomic profile enriched for cytotoxic T-cell signatures (e.g., Tc17 cells), and a dual skin–joint inflammatory circuit. Recognizing these molecular distinctions is not merely an academic exercise but is essential for developing the next generation of precision diagnostics and targeted therapies tailored to the specific immunopathology of each condition.

Looking ahead, overcoming these challenges requires collaborative efforts across multiple stakeholders. Rigorous large-scale clinical studies must be conducted to validate the robustness and clinical utility of biomarkers. Interdisciplinary collaboration—integrating rheumatology, bioinformatics, statistics, and laboratory medicine—is essential for developing reliable analytical workflows and interpretation standards. Innovative clinical trial designs, such as biomarker-guided patient enrichment strategies, can accelerate the evaluation and approval of targeted therapies. Furthermore, technological advances like single-cell sequencing, spatial omics, and liquid biopsies will continue delivering higher-resolution data, driving the discovery of more refined disease subtypes.

Ultimately, the successful integration of multi-omics into clinical practice will depend as much on addressing ethical, social, and accessibility challenges as on scientific progress. Protecting patient data, ensuring equitable access to advanced diagnostics, and demonstrating cost-effectiveness are not afterthoughts, but integral components of this translational pathway. Through sustained, interdisciplinary collaboration and rigorous validation, a multi-omics-guided approach holds the promise to fundamentally improve outcomes and quality of life for every patient across the spondyloarthritis spectrum.
